# Comparing Older Adults Assigned a Low Acuity Triage Score to Their Younger Counterparts in the Emergency Department: A Review of Patient Characteristics and Outcomes

**DOI:** 10.7759/cureus.102649

**Published:** 2026-01-30

**Authors:** Kayla Furlong, Rachel Price, Victoria Brannan, Augustine J Devasahayam, Susan Mercer, Yanqing Yi, Peter Rogers, Kimberly Babb, Michael H Parsons

**Affiliations:** 1 Department of Emergency Medicine, Memorial University of Newfoundland, St. John's, CAN; 2 Faculty of Medicine, Memorial University of Newfoundland, St. John's, CAN; 3 Department of Research and Innovation, Newfoundland and Labrador Health Services, St. John's, CAN; 4 Department of Geriatric Medicine, Newfoundland and Labrador Health Services, St. John's, CAN

**Keywords:** emergency department, geriatric, older adults, resource utilization, undertriage

## Abstract

Background

Older adults (≥65 years) present to the emergency department (ED) more frequently and have longer lengths of stay in the ED when compared to their younger counterparts. Older adults are also at risk for undertriage in the ED and may require hospital admission or further intervention despite having a “low acuity” triage score. Our study aimed to describe and compare the characteristics, resource utilization, and health outcomes of older adults with “low acuity” triage scores to their younger counterparts in urban EDs in Newfoundland and Labrador (NL).

Methods

A chart review was performed on older adults (≥65 years) assigned a “low acuity” triage score, defined by the Canadian Triage and Acuity Scale (CTAS) as 4 or 5. Patients aged 40-55 years were selected as controls, serving as younger counterparts to the older adults. The primary outcome was hospital admission at the index ED visit. Secondary outcomes included length of stay in the ED and 14-day ED re-visit rate, among others. Subgroups included those 65-74 years, 75-84 years, and 85 years and older.

Results

Eight hundred fifty-one (n=851) patients were screened, and 554 were included. The mean age was 78.5 and 46.8 years, respectively, for older adults and controls. Older adults were more likely to arrive by ambulance (19.7% versus 4.8%, p=0.001) and require a social work consultation (5% versus 0%, p=0.028). Hospital admission did not differ between older adults and controls (1.1% versus 0%, p>0.05). Among subgroups, those 85 years and older were more likely to require more testing and interventions, social support services, and hospital admission compared to those <85 years (p<0.05 for all).

Conclusions

Older adults were more likely to require ambulance services and a social work consultation compared to their younger counterparts. Among older adults, the use of ambulance services, ED resources, and hospital admission was highest among those 85 years and older. The urban EDs in NL should optimize ambulance utilization, expand access to social work and interdisciplinary support, and allocate resources to address the needs of the oldest-old. Implementing targeted care pathways, such as managing musculoskeletal patients with low-acuity CTAS scores through advanced practice providers, may enhance efficiency and reduce unnecessary admissions. Further research is needed to assess the health and economic impact of these strategies and to guide evidence-based planning for older adult care in the EDs.

## Introduction

Older adults (≥65 years) represent the largest proportion of adults (24.2%) presenting to emergency departments (EDs) in Canada [1]. Currently, 19% of Canadian adults fall into this age group, a figure projected to rise to approximately one in four by 2036, with some regions reaching as high as one in three [2]. Compared to their younger counterparts, older adults exhibit distinct physiological differences and increased multimorbidity. In the ED, they present more frequently, experience longer lengths of stay and delayed treatment, and have higher admission and re-presentation rates, often with adverse events after an ED visit [3].

The Canadian Triage and Acuity Scale (CTAS) is a five-level numerical scale widely used in Canadian EDs to prioritize patients based on the severity and urgency of their condition [4]. While not designed to predict outcomes, CTAS has been correlated with resource utilization and various health outcomes, including the use of ambulance services, diagnostic imaging, laboratory testing, specialist consultation, hospital costs, hospital admission, and disposition [5]. Research suggests that older adults are undertriaged, leading to delays in treatment, inadequate care, and potential deterioration while waiting, and would benefit from a higher triage score to expedite care and improve health outcomes [6]. In a UK study of older adults, those aged 85 and above experienced the longest ED waits, with 39.7% (5,098/12,833) remaining in the department for more than eight hours [7]. They also had the highest inpatient admission rate, with 70% requiring admission, significantly more than adults aged 18-64, 65-74, and 75-84 [7]. Such a misclassification of triage level can result in adverse consequences, including increased morbidity and mortality [8]. However, advancing age, regardless of triage level, has been associated with higher resource utilization, increased hospital admissions, and greater mortality when comparing older adults to their younger counterparts [9]. Even when assigned a “low acuity” CTAS score (4 or 5), one study reports higher rates of ED resource utilization, hospital admission, and admission on re-presentation in older adults compared to their younger counterparts [10].

There is a need to further understand the characteristics, resource utilization, and outcomes of “low acuity” older adults, particularly the “oldest-old” (≥85 years), a rapidly growing demographic in Canada [11]. This group has been shown to have longer ED stays, higher admission rates, and increased mortality compared to those aged 65-74 years [12]. Additionally, there is a paucity of research examining ED outcomes among older adults in Newfoundland and Labrador (NL), the Canadian province with the highest proportion and fastest growth rate of older adults [2]. Our study aimed to analyze patient characteristics, resource utilization, and health outcomes among older adults aged ≥65 years presenting to the EDs in NL with a “low acuity” CTAS score (4 or 5) and compare them to younger counterparts aged 40-55 years with an a priori subgroup analysis of the 65-74 years (youngest-old), 75-84 years (middle-old), and 85 years and older (oldest-old) groups.

## Materials and methods

Study design, setting, and time period

This was a retrospective cohort study conducted in the two EDs of the Eastern Urban Zone of NL (Health Sciences Centre and St. Clare’s Mercy Hospital) in St. John’s (both affiliated with Memorial University). These are the only hospitals with full-service EDs in the zone, serving as the primary emergency care centers and capturing all local ED visits. The study period spanned from July 1, 2019, to September 30, 2019, during which 24,382 ED visits were recorded (Health Sciences Centre: 14,290, St. Clare’s Mercy Hospital: 10,092). In 2018, older adults comprised 28.2% of total ED visits to these two sites (Health Sciences Centre: 27%, St. Clare’s Mercy Hospital: 30%). The NL Health Research Ethics Board (HREB) approved this study (#2021.0333; date: June 18, 2020) according to the Tri-Council Policy Statement: Ethical Conduct for Research Involving Humans (TCPS2) and the ICH-Good Clinical Practice Guidelines (ICH-GCP). The NL HREB waived consent for this study.

Population

**Figure 1:**
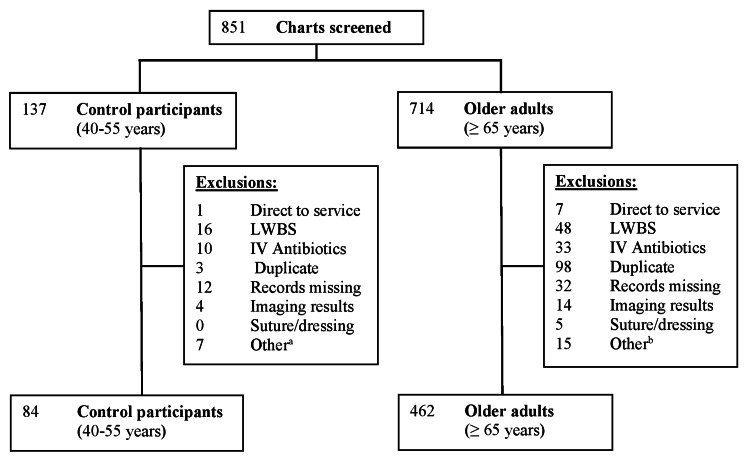
Flowchart of screening and enrollment for older adults and control participants ^a^Other reasons included nursing assessment only (n=1), prescription refill (n=2), patient of another health authority (n=1), and wound or bloodwork re-check (n=3) ^b^Other reasons included blood transfusion (n=2), cast check (n=3), nursing assessment only (n=4), urology technologist only (n=3), and wound care re-check (n=3) IV: intravenous, LWBS: leaving without being seen

Patients aged ≥65 years with a “low acuity” CTAS score (4 or 5) were included in the older adult cohort. Patients aged 40-55 years were selected as control participants, serving as younger counterparts to the older adults. One unique ED visit per patient was included and coined the index visit. Each patient’s index visit was randomly selected if multiple visits occurred during the study period. We decided on a priori subgroups of age, including (1) 65-74 years (youngest-old), (2) 75-84 years (middle-old), and (3) 85 years and older (oldest-old). Exclusion criteria included direct referral to a specialty service (e.g., internal medicine), leaving without being seen, returning for intravenous (IV) antibiotics, cast or wound checks, routine bloodwork, blood transfusions, prescription refills, or missing data (Figure 1 and Supplemental Figure 1).

Data collection

Trained data abstractors (KF and RP), familiar with the electronic health information system, reviewed demographic and clinical data using standardized extraction forms. Reviewed documents included triage records, nursing and physician notes, emergency medical services notes, consultation and admission notes, laboratory and imaging records, and social work documentation. Documentation from the HomeFirst program, a short-term post-ED multidisciplinary support service for older adults who require assistance for up to 30 days after an ED visit, was also reviewed. Visit history was assessed up to six months prior to and 14 days post-index ED visit. ED documentation, consultation, and admission notes were reviewed up to two weeks after the index visit.

Demographic variables collected included age, sex, postal code, current place of residence (e.g., home, personal care home, long-term care, etc.), presenting complaint, triage location, family physician listed, and past medical history. A personal care home is a residence that provides lodging, meals, and assistance with activities of daily living, known in some areas as assisted living facilities. Participant postal codes were used to define rurality according to Canada Post guidelines, where the second character of the Forward Sortation Area distinguishes urban (1-9) from rural (0) locations. ED variables collected included CTAS, triage vitals, re-triage, time of assessment and discharge, testing and interventions, consultations, discharge diagnosis, and discharge disposition. Inter-rater reliability was conducted by a different reviewer (VB) for 20% of charts (n=113), with discrepancies resolved via discussion and consensus. As an additional inter-rater reliability measure, we assigned CTAS scores to 20% of charts (n=113). One reviewer performed this task, who was trained in CTAS scoring (VB).

Outcome measures

Our primary outcome was admission to the hospital at the ED index visit. Secondary outcomes included ED length of stay, 14-day ED re-visit rate, reason for re-visit, 14-day admission rate, whether level of care increased at discharge, and ED visits and admissions in the prior six months.

Sample size

The primary hypothesis that older adults (≥65 years) presenting with low-acuity conditions in the ED would have higher hospital admission rates than their younger counterparts guided our sample size calculation. Based on an estimated admission rate of 8.9% in patients 85 years and older [10], where significance is set at 0.05 and statistical power at 80%, we calculated that 161 patients aged 85 years and older (oldest-old) were required, with a control group of 84 patients. With this estimation, we assumed that we would capture enough patients in the 65-74 years (youngest-old) and 75-84 years (middle-old) subgroups to have the power to detect differences in those groups, as the proportion of patients is likely to be higher in these two age groups based on the age demographics in NL [13]. We therefore aimed to include 84 patients in our control group and 483 in our older adult cohorts (161 per subgroup) for a total sample size of 567 for the primary outcome, which would provide sufficient power for the analysis.

Data analysis

Means with standard deviation (SD) and proportions with confidence intervals (CIs) are reported for continuous and categorical variables, respectively. Median and range are reported for non-normally distributed data. Student’s t, chi-square, and one-way analysis of variance (ANOVA) tests were used to compare normally distributed variables. Mann-Whitney U and Kruskal-Wallis tests were used to compare non-normally distributed variables. Cohen’s kappa was calculated for inter-rater reliability for CTAS scores. The assigned CTAS scores were not included in the main analysis and were used only for inter-rater reliability. A stratified analysis using our a priori age subgroups was performed for all outcomes. A significance level was set at <0.05. Data were analyzed using SPSS software (version 28.0.1.0) (IBM Corp., Armonk, NY).

## Results

Patient characteristics and resource utilization

A total of 851 patients (714 older adults and 137 controls) were screened against our inclusion criteria, and 546 were included (462 older adults and 84 controls; Figure 1). The average age was 78.5 years for older adults and 46.8 years for controls. Older adults were more likely to arrive by ambulance (19.7% versus 4.8%, p=0.001; Table 1) and not from their own home (93.9% versus 98.8%, p=0.03; Table 1). Older adults presented more frequently with a fall/mobility issue (7.6% versus 1.2%) and genitourinary complaints (7.6% versus 6%, p=0.03; Table 1). Older adults were more likely to have chronic diseases, such as diabetes, hypertension, and heart disease (p<0.0001; Table 1). Abnormal triage vitals were not common in either group (Table 1).

**Table 1:** Patient characteristics of older adults and controls Missing data, controls: triage location (n=22); missing data, older adults: triage location (n=80) ^a^Inpatient unit from a psychiatric facility ^b^Unknown location *p<0.05 CTAS: Canadian Triage and Acuity Scale, SD: standard deviation

Characteristics	Controls (n=84)	Older adults (n=462)
Age (years), mean (SD)	46.8 (4.4)	78.5 (8.5)
Gender (female), number (%)	48 (57.1)	285 (61.7)
Rurality, number (%)		
Urban	74 (88.1)	387 (83.8)
Rural	10 (11.9)	75 (16.2)
Arrival mode*, number (%)		
Ambulance	4 (4.8)	91 (19.7)
Self-presented	80 (95.2)	371 (80.3)
Residence*, number (%)		
From home	83 (98.8)	434 (93.9)
From personal care home	0 (0)	21 (4.5)
From long-term care	0 (0)	6 (1.3)
Other	1^a^ (1.2)	1^b ^(0.2)
CTAS score on presentation, number (%)		
4	78 (92.9)	445 (96.3)
5	6 (7.1)	17 (3.7)
Triage location, number (%)		
Ambulatory care	52 (61.9)	296 (64.1)
Bedded unit	10 (11.9)	86 (18.6)
Family physician listed, number (%)	79 (94)	446 (96.5)
Reasons for visit*, number (%)		
Musculoskeletal	31 (36.9)	155 (33.5)
Cardiorespiratory	11 (13.1)	20 (4.3)
Head, neck, and ENT	9 (10.7)	40 (8.6)
Back pain	8 (9.5)	27 (5.8)
Abdominal pain	5 (6)	22 (4.8)
Genitourinary	5 (6)	35 (7.6)
Fall/mobility issues	1 (1.2)	35 (7.6)
Medical device problem	0 (0)	8 (1.7)
Social issues	0 (0)	4 (0.9)
Past medical history*, number (%)		
Diabetes	12 (14.3)	92 (20)
Hypertension	16 (19)	237 (51.3)
Depression/anxiety	16 (19)	55 (12)
Heart disease	14 (16.7)	231 (50)
Atrial fibrillation	1 (1.2)	40 (8.7)
Chronic obstructive pulmonary disease	1 (1.2)	40 (8.7)
Osteoarthritis	1 (1.2)	51 (11)
Dementia	0 (0)	21 (4.5)
Abnormal triage vitals, number (%)		
Temperature > 38°C	0 (0)	0 (0)
Heart rate > 100	10 (11.9)	44 (9.5)
Respiratory rate > 20	0 (0)	25 (5.4)
Systolic blood pressure < 90 mmHg	0 (0)	0 (0)
Oxygen saturation < 90%	0 (0)	4 (0.9)
Glucose < 4 mmol/L	0 (0)	0 (0)

Older adults were not more likely to be re-triaged (0.9% versus 0%, p=0.51; Table 2). Diagnostic testing, investigation, and consultation rates were similar between older adults and controls (p>0.05 for all; Table 2). Older adults were more likely to require a social work consultation (5% versus 0%, p=0.028; Table 2). Discharge diagnosis did not differ between groups (p=0.28; Table 3).

**Table 2:** Emergency department variables of older adults and controls Missing data, controls: time to assessment (n=1), length of stay in the ED (n=2) Missing data, older adults: time of assessment (n=1), time to assessment (n=9), length of stay in the ED (n=2) ^a^Other consults in the ED, controls: gynecology (n=1), neurosurgery (n=2), oral surgery (n=1), orthopedics (n=3), plastic surgery (n=5), rheumatology (n=1) ^b^Other consults in the ED, older adults: community health (n=1), dermatology (n=2), ENT (n=4), gastroenterology (n=1), interventional radiology (n=1), neurology (n=1), neurosurgery (n=1), ophthalmology (n=4), orthopedics (n=30), plastic surgery (n=5), psychiatry (n=1), urology (n=12), vascular surgery (n=1) ^c^Transferred to psychiatric assessment unit *p<0.05 CT: computed tomography, ED: emergency department, IV: intravenous, SD: standard deviation

Variables	Controls (n=84)	Older adults (n=462)
Emergency department, number (%)		
Health Sciences Centre	40 (47.6)	208 (45)
St. Clare’s Mercy Hospital	44 (52.4)	254 (55)
Re-triage to more acute setting, number (%)	0 (0)	4 (0.9)
Time to assessment (hours), mean (SD)	2.7 (2)	2.4 (2)
X-ray performed, number (%)	33 (39.3)	176 (38.1)
Bloodwork drawn, number (%)	6 (7.1)	95 (20.6)
CT scan performed, number (%)	6 (7.1)	54 (11.7)
IV fluids given, number (%)	1 (1.2)	14 (3)
IV antibiotics given, number (%)	3 (3.6)	11 (2.4)
Consult in the ED, number (%)	13 (15.5)	76 (16.5)
Medicine	0 (0)	10 (2.2)
Surgery	0 (0)	1 (0.6)
Geriatrics	0 (0)	1 (0.2)
Other^a^	13^a^ (100)	64^b^ (84.2)
Consults for inpatient services, number (%)	0 (0)	14 (3)
Other services used in the ED, number (%)		
Social Work*	0 (0)	23 (5)
HomeFirst Program	0 (0)	11 (2.4)
Discharge diagnosis, number (%)		
Soft tissue/extremity injury	25 (29.8)	117 (25.3)
Fracture	4 (4.8)	32 (6.9)
Back pain	4 (4.8)	30 (6.5)
Urinary tract infection	3 (3.6)	33 (7.1)
Respiratory tract infection	7 (8.3)	24 (5.2)
Cellulitis/rash/abscess/rash	5 (6)	28 (6.1)
Laceration/bite/foreign body	6 (7.1)	24 (5.2)
Disposition, number (%)		
Home	84 (100)	420 (90.9)
Home with supports	0 (0)	13 (2.8)
Personal care home	0 (0)	16 (3.5)
Long-term care	0 (0)	7 (1.5)
Other	0 (0)	1^c^ (0.2)

**Table 3:** Outcome measures of older adults and controls Missing data, older adults: length of stay (n=2) ED: emergency department, SD: standard deviation

Outcomes	Controls (n=84)	Older adults (n=462)	P value
Admitted, number (%)	0 (0)	5 (1.1)	0.43
Length of ED stay (hours), mean (SD)	3.8 (2.3)	3.9 (3)	0.22
14-day ED re-visit rate, number (%)	11 (13.1)	71 (15.4)	0.59
Reason for re-visit the same, number (%)	8 (72.3)	35 (49.3)	0.15
Disposition where level of care increased, number (%)	0 (0)	18 (3.9)	0.047
14-day admission rate, number (%)	0 (0)	7 (1.5)	0.20
ED visits in the past 6 months, median (range)	0 (0-36)	0 (0-17)	0.049
Hospital admissions in the past 6 months, median (range)	0 (0-2)	0 (0-4)	0.03

Outcome analysis

There was no difference in hospital admission rate between older adults (1.1%) and controls (0%, p = 0.43; Table 3). For secondary outcomes, older adults had more ED visits (p=0.049) and hospital admissions in the prior six months (p=0.03; Table 3). Older adults were also more likely to have an increased level of care needs at ED discharge (3.9% versus 0%, p=0.047; Table 3). There were no statistically significant differences for length of stay in the ED, 14-day ED re-visit rate, reason for re-visit, and 14-day admission rate (p>0.05 for all; Table 3).

Subgroup analysis

Supplemental Figure 1 describes the participant recruitment flow for all subgroups. Among subgroups, those 85 years and older (oldest-old) were more likely to present with falls/mobility (13.6% versus 8.1% versus 6.2%) and social (2.1% versus 0.6% versus 0%) issues compared to those 75-84 years (middle-old) and 65-74 years (youngest-old, p=0.013; Supplemental Table 1). Those 85 years and older (oldest-old) were also more likely to arrive by ambulance (40.7% versus 14.9% versus 6.2%), be triaged in a bedded unit (29.3% versus 19.2% versus 8.6%), require IV fluids (5.7% versus 3.1% versus 0.6%), IV antibiotics (5% versus 0.6% versus 1.9%), a consultation for inpatient services (29.6% versus 24% versus 0%), a social work consultation (9.3% versus 4.3% versus 1.2%), and HomeFirst services (5% versus 2.5% versus 0%, p<0.05, Supplemental Table 1 and Table 2). Those 85 years and older (2.9%) were more likely to be admitted than those 65-74 years (0.6%) and 75-84 years (0%, p=0.045). Those 85 years and older were also more likely not to return home (77.8% versus 93.8% versus 100%, p<0.05; Supplemental Table 2 and Table 3).

Inter-rater reliability

Among the reviewed charts, discrepancies were observed for 150 data points (2% of all data points reviewed), and all were resolved by discussion and consensus. The most common reason for discrepancy was interpretation of penmanship (21%). For CTAS scores, 29 scores differed (25.7%). Twenty-three (79%) discrepant scores were up-triaged (i.e., 5 to 4, 4 to 3, 5 to 3, etc.). Five (17%) discrepant scores were down-triaged (i.e., 4 to 5). The unweighted kappa value was 0.41 (95% CI: 0.32-0.499).

## Discussion

Older adults classified as “low acuity” at triage required more resources than their younger counterparts, particularly for ambulance and ED services in NL. Approximately one in five (19.7%) older adults arrived via ambulance, with rates nearly one in two (40.7%) among those 85 years and older (oldest-old). Those 85 years and older (oldest-old) required more interventions and resources. Those 85 years and older (oldest-old) were most dependent on ED resources, requiring IV fluids (5%), IV antibiotics (5.7%), and a social work consultation (9.3%). While overall admission rates were low among older adults (1.1%), they increased with age, peaking at 2.9% in those 85 years and older (oldest-old). No patients <75 years were admitted.

The high costs of ambulance services on the healthcare system are previously established [12]. In our study, using data from 2019 in NL across two EDs, older adults (≥65 years, n=462) were more likely to arrive by ambulance than younger adults (19.7% versus 4.8% in those aged 40-55) (Table 1). This is consistent with a 2012-2013 study in Nova Scotia, where 32.4% (11,173/34,461) of older adults across four EDs arrived by ambulance, indicating a broader trend of higher ambulance use among older adults in Atlantic Canada [11]. Ambulance use also increased markedly with age in our cohort: 6.2% of the youngest-old (65-74 years), 14.9% of the middle-old (75-84 years), and 40.7% of the oldest-old (≥85 years) arrived by ambulance (Table 1). A similar age-related pattern was observed in Nova Scotia, with 23.1%, 34.3%, and 52.9% of the youngest-, middle-, and oldest-old, respectively, arriving by ambulance [11]. Furthermore, findings highlight that older adults (≥65 years) represent a substantial proportion of emergency medical services demand (close to 50% of the emergency call volume), with the majority (87.7%) of these calls resulting in ambulance transport [14]. It is notable that older adults generally use ambulance services at high rates (>50%) compared to their younger counterparts [15], even if the reason is less urgent than what is typically defined as “an emergency” [16,17]. Both the rate of response and transfer to the ED appear to be consistent in the literature and highest among the oldest-old, contributing to higher ambulance service costs [12,14].

Admission rate (1.1%) is lower in NL than recently reported rates of “low acuity” older adults (4.6%-6.1%) in other Canadian jurisdictions [11] and lower than one study reporting admission rates of 19% among CTAS 4 older adults and 4.3% among CTAS 5 [5]. One Canadian study does report an admission rate of 1.6% in older adults with CTAS scores of 5 [18]. However, the majority (92.9%) of our “low acuity” scores were CTAS 4. In those 85 years and older (oldest-old), our admission rate was 2.9%. This is much lower than the 8.9% reported rate for the oldest-old in Ontario in a similar study [10]. Although not known, we hypothesize that the use of the CTAS frailty modifier [19] or clinical gestalt of our nursing staff may have accounted for inherent up-triage to CTAS 3 from 4 or 5. Agreement between CTAS scores was similar to Göransson et al. [20], although lower than some others [10,18].

Many older adults present atypically or with non-specific complaints due to changing physiology, multimorbidity, or polypharmacy, which can lead to undertriage [21]. However, atypically presenting older adults often have serious medical issues requiring comprehensive workups [21,22]. A recent report indicates that ED visit costs generally increase with age (e.g., 65 years and older: $1,100 USD per visit versus 18-64 years: $660-880 USD per visit) [23]. Older adults arrived at our EDs more frequently with complaints of falls or mobility issues (7.9%) than their younger counterparts. In 2018, the direct cost of fall-related injuries among Canadian adults 65 years and older was $5.6 billion, double that of injuries in those 25-64 years [24]. Health systems utilization beyond the ED, including hospital admission and re-visit rates, was not different in our study but has been previously observed to be higher in older adults elsewhere [25-27].

Our research has implications for triage tools, the flow of older adults, resource utilization, and health outcomes in the EDs in NL. These findings underscore the need for triage modifications incorporating age or frailty. Such modifiers of CTAS exist [19]; however, their use and association with resource utilization and health outcomes do not appear to be documented in the literature. A study using the Emergency Severity Index (ESI), a triage tool commonly used in the United States and elsewhere, demonstrated that age, independent of the ESI, identifies older adults with higher ED resource use, hospital admission, and mortality compared to their younger counterparts [9]. The use of the Clinical Frailty Scale (CFS) in the ED is also well established and can help guide care plans and decision-making among older adults [28]. Adding CFS to triage assessments in “low acuity” older adults identifies the need for ICU admission [28]. Another study, using a frailty index, demonstrated that the frailty and acuity both predicted hospital admission but that frailty additionally predicted future hospitalization and ED return visits [29].

The EDs in NL should prepare for older adults and their need for more comprehensive workups and social support services, even if placed in a “low acuity” triage category. Future research studies may seek to determine the relationship between frailty, acuity, resource utilization, and health outcomes in this “low acuity” population. More research and replicability are required, particularly in those 85 years and older (oldest-old), as they seem to be at the highest risk for undertriage and resource utilization. The benefits and cost-savings of interdisciplinary assessment in older adults with “low acuity” triage scores, particularly among those 85 years and older (oldest-old), could also be explored.

Strengths and limitations

The strengths of our study include the focus on “lower acuity” older adults in the two urban EDs in NL. It is recognized that older adults are at risk of undertriage [6], and if undertriaged, they would fall into a “low acuity” category. This is the first study of its kind to examine “low acuity” older adults in NL, the province with the highest proportion and rate of growth of older adults in Canada [2]. To mitigate misclassification bias, 20% of charts were reviewed. Discrepancies were found for only a trivial amount (2%) of variables reviewed, and all were resolved via consensus.

Our study has a few limitations. This study was conducted at two urban EDs in one health region in NL, and our findings may not generalize to rural settings or other provinces with different healthcare systems. Potential biases include selection bias, as only two urban EDs were included, and information bias due to the retrospective chart review design. To address these, we included all eligible patients from both sites, used standardized data extraction procedures, and cross-checked key variables to ensure accuracy and completeness of the dataset. We have reported missing data for some variables that had hand-written documentation (i.e., triage location, time of assessment, length of ED stay, etc.), although missing information was trivial (n=1 to 9) for most variables, except for triage location (n=102).

We aimed to ensure sufficient power to detect differences in the ≥85 years (oldest-old) subgroup, a population underrepresented in previous studies; however, we fell slightly short of the target sample for this subgroup (n=138/161). All eligible patients were reviewed for inclusion; however, there were no more patients who were 85 years and older with a CTAS 4 or 5 during the study period. The slight underpowering may increase the risk of type II error but reflects the natural patient distribution rather than a sampling limitation. Although subgroup comparisons suggested that adults aged 85 years and older had higher hospital admission rates and were more likely not to return home, the very low number of events renders these findings statistically unstable, and they should therefore be interpreted as descriptive trends rather than definitive results. Lastly, our CTAS scorer was not blinded to study outcomes, which may have led to the 79% up-triage rate.

## Conclusions

Older adults were significantly more likely to arrive by ambulance and require social work involvement than their younger counterparts, whereas hospital admission rates did not differ meaningfully between groups. Among older adults, those aged 85 years and above (oldest-old) were significantly more likely to require additional testing and interventions, social support services, and hospital admission compared with those under 85. Use of ambulance services, ED resources, and hospital admission was highest in this oldest subgroup. Given the projected increase in this population, ED systems should adapt to their unique needs, including evaluation of ambulance services, enhanced triage protocols, and expanded interdisciplinary support services. Further research is needed to optimize triage accuracy and healthcare resource allocation for older adults in the ED setting. Larger studies are required to strengthen the associations found here and explore health and economic outcomes in this population further.

## Figures and Tables

**Figure 2:**
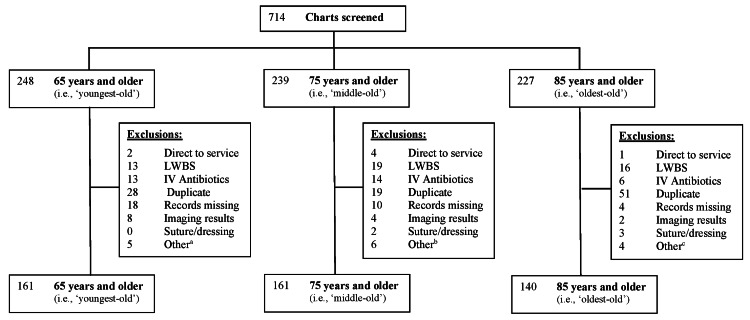
Flowchart of screening and enrollment for older adults stratified by age subgroups ^a^Other reasons included nursing assessment only (n=2) and would care re-check (n=3) ^b^Other reasons included blood transfusion (n=1), cast check (n=2), nursing assessment only (n=1), and urology technologist only (n=2) ^c^Other reasons included blood transfusion (n=1), cast check (n=1), nursing assessment only (n=1), and urology technologist only (n=1) IV: intravenous, LWBS: leaving without being seen

## Appendices

Supplemental Figure 1

Figure 2 presents the participant recruitment flow for all subgroups.

Supplemental Table 1

Table 4 presents the characteristics of older adult patients by subgroup. 

**Table 4:** Older adult patient characteristics by subgroup Missing data, 65-74 years: triage location (n=31) Missing data, 75-84 years: triage location (n=30) Missing data, 85 years and older: triage location (n=19) ^a^Unknown location (n=1) *p<0.05 SD: standard deviation

Characteristics	Youngest-old (65-74 years) (n=161)	Middle-old (75-84 years) (n=161)	Oldest-old (85 years and older) (n=140)
Age (years), mean (SD)	69.1 (2.9)	78.8 (2.7)	88.8 (3.7)
Gender (female), number (%)	91 (56.5)	101 (62.7)	93 (66)
Rurality, number (%)			
Urban	129 (80.1)	138 (85.7)	120 (85.7)
Rural	32(19.9)	23 (14.3)	20 (14.3)
Arrival mode, number (%)			
Ambulance	10 (6.2)	24 (14.9)	57 (40.7)
Self-presented	151 (93.8)	137 (85.1)	83 (59.3)
Residence, number (%)			
From home	159 (98.8)	156 (96.9)	119 (85)
From personal care home	1 (0.6)	4 (2.5)	16 (11.4)
From long-term care	0 (0)	1 (0.6)	5 (3.6)
Other	1^a^ (0.6)	0 (0)	0 (0)
CTAS score on presentation, number (%)			
4	152 (94.4)	157 (97.5)	136 (97.1)
5	9 (5.6)	4 (2.5)	4 (2.9)
Triage location, number (%)			
Ambulatory care	116 (72)	100 (62.2)	80 (57.1)
Bedded unit	14 (8.6)	31 (19.2)	41 (29.3)
Family physician listed, number (%)	152 (94.4)	161 (100)	133 (95)
Reasons for visit, number (%)			
Musculoskeletal	62 (38.5)	55 (34.2)	38 (27.1)
Cardiorespiratory	4 (2.5)	14 (8.7)	6 (4.3)
Head, neck, and ENT	12 (7.5)	16 (9.9)	12 (8.6)
Back pain	10 (6.2)	6 (3.7)	11 (7.9)
Abdominal pain	11 (6.8)	8 (5)	3 (2.1)
Genitourinary	10 (6.2)	13 (8.1)	12 (8.6)
Fall/mobility issues	7 (4.3)	9 (5.6)	19 (13.6)
Medical device problem	3 (1.9)	4 (2.5)	1 (0.7)
Social issues	0 (0)	1 (0.6)	3 (2.1)
Past medical history, number (%)			
Diabetes	30 (18.6)	35 (21.7)	27 (19.3)
Hypertension	64 (39.8)	89 (55.3)	84 (60)
Depression/anxiety	17 (10.6)	22 (13.7)	16 (11.4)
Dementia	2 (1.2)	6 (3.7)	13 (9.3)
Atrial fibrillation	3 (1.9)	18 (11.2)	19 (13.6)
Heart disease/dyslipidemia	77 (47.8)	94 (58.4)	60 (42.9)
Chronic obstructive pulmonary disease	6 (3.7)	9 (5.6)	10 (7.1)
Osteoarthritis	4 (2.5)	14 (8.7)	33 (23.6)
Abnormal triage vitals, number (%)			
Temperature > 38°C	0 (0)	0 (0)	0 (0)
Heart rate > 100	10 (6.2)	18 (11.2)	16 (11.4)
Respiratory rate > 20	6 (3.8)	9 (5.6)	10 (7.1)
Systolic blood pressure < 90 mmHg	0 (0)	0 (0)	0 (0)
Oxygen saturation < 90%	1 (0.6)	3 (1.9)	0 (0)
Glucose < 4 mmol/L	0 (0)	0 (0)	0 (0)

Supplemental Table 2

Table 5 presents older adult emergency department variables by subgroup.

**Table 5:** Older adult emergency department variables by subgroup Missing data, 75-84 years: time to assessment (n=8) Missing data, 85 years and older: time to assessment (n=1) ^a^Other consults in the ED, 65-74 yrs: dermatology (n=2), neurosurgery (n=1), ophthalmology (n=1), orthopedics (n=13), plastic surgery (n=3), urology (n=4) ^b^Other consults in the ED, 75-84 years: ENT (n=3), interventional radiology (n=1), ophthalmology (n=1), orthopedics (n=10), plastic surgery (n=2), psychiatry (n=1), urology (n=4) ^c^Other consults in the ED, 85 years and older: community health (n=1), ENT (n=1), gastroenterology (n=1), neurology (n=1), ophthalmology (n=2), orthopedics (n=7), urology (n=4), vascular surgery (n=1) ^d^Transferred to psychiatric assessment unit *p<0.05 CT: computed tomography, ED: emergency department, IV: intravenous, SD: standard deviation

Variables	Youngest-old (65-74 years) (n=161)	Middle-old (75-84 years) (n=161)	Oldest-old (85 years and older) (n=140)
Emergency department, number (%)			
Health Sciences Centre	68 (42.2)	72 (44.7)	68 (48.6)
St. Clare’s Mercy Hospital	93 (57.8)	89 (55.3)	72 (51.4)
Re-triage to more acute setting, number (%)	0 (0)	1 (0.6)	3 (2.1)
Time to assessment (hours), mean (SD)	2.5 (2.1)	2.5 (2.1)	2.2 (1.9)
X-ray performed, number (%)	57 (35.4)	57 (35.4)	62 (44.3)
Bloodwork drawn, number (%)	4 (2.5)	44 (27.3)	47 (33.6)
CT scan performed, number (%)	36 (22.4)	9 (5.6)	9 (6.4)
IV fluids given*, number (%)	1 (0.6)	5 (3.1)	8 (5.7)
IV antibiotics given*, number (%)	3 (1.9)	1 (0.6)	7 (5)
Consult in the ED, number (%)	24 (14.9)	25 (15.5)	27 (9.3)
Medicine	0 (0)	3 (1.9)	7 (26)
Surgery	0 (0)	0 (0)	1 (3.7)
Geriatrics	0 (0)	0 (0)	1 (3.7)
Other	24^a^ (100)	22^b^ (13.7)	18^c^ (66.7)
Consults for inpatient services*, number (%)	0 (0)	6 (24)	8 (29.6)
Other services used in the ED, number (%)			
Social Work*	2 (1.2)	7 (4.3)	13 (9.3)
HomeFirst Program*	0 (0)	4 (2.5)	7 (5)
Discharge diagnosis, number (%)			
Soft tissue/extremity injury	38 (23.6)	39 (24.2)	40 (28.6)
Fracture	12 (7.5)	10 (6.2)	10 (7.1)
Back pain	11 (2.4)	6 (3.7)	13 (9.3)
Urinary tract infection	9 (5.6)	13 (8.1)	11 (7.9)
Respiratory tract infection	4 (2.5)	15 (9.3)	5 (3.6)
Cellulitis/rash/abscess/rash	7 (4.3)	15 (9.3)	6 (4.3)
Laceration/bite/foreign body	12 (7.5)	6 (3.7)	6 (4.3)
Disposition*, number (%)			
Home	161 (100)	151 (93.8)	108 (77.8)
Home with supports	0 (0)	4 (2.5)	9 (6.4)
Personal care home	0 (0)	3 (1.9)	13 (9.3)
Long-term care	0 (0)	1 (0.6)	6 (4.3)
Other	0 (0)	1^d^ (0.6)	0 (0)

Supplemental Table 3

Table 6 presents the outcome measures of older adults by subgroup.

**Table 6:** Outcome measures of older adults by subgroup Missing data, 75-84 years: length of stay (n=2) ED: emergency department, SD: standard deviation

Outcomes	Youngest-old (65-74 years) (n=161)	Middle-old (75-84 years) (n=161)	Oldest-old (85 years and older) (n=140)	P value
Admitted, number (%)	0 (0)	1 (0.6)	4 (2.9)	0.045
Length of ED stay (hours), mean (SD)	3.6 (2.5)	3.9 (2.8)	3.6 (3.1)	0.21
14-day ED re-visit rate, number (%)	22 (13.7)	32 (19.9)	17 (12.1)	0.14
Reason for re-visit the same, number (%)	12 (54.5)	15 (46.9)	8 (47.1)	0.84
Disposition where level of care increased, number (%)	0 (0)	6 (3.7)	12 (8.6)	0.077
14-day admission rate, number (%)	0 (0)	5 (3.1)	2 (1.4)	0.085
ED visits in the past 6 months, median (range)	0 (0-15)	0 (0-17)	1 (0-6)	0.066
Hospital admissions in the past 6 months, median (range)	0 (0-3)	0 (0-4)	0 (0-4)	0.27
